# Supplementary material for: The DNA adenine methylase of Salmonella Enteritidis promotes their intracellular replication by inhibiting arachidonic acid metabolism pathway in macrophages
Source: Front Microbiol. 2023 Mar 2;14:1080851. doi: 10.3389/fmicb.2023.1080851 (PMC10018194; doi:10.3389/fmicb.2023.1080851)
Supplement: Supplementary file 2 [file Data_Sheet_2.zip › S1 Appendix. Non-targeted metabolomics raw data/4.MetDiffAnalysis/C50336_WT.vs.control/C50336_WT.vs.control_pos_corr.pdf]

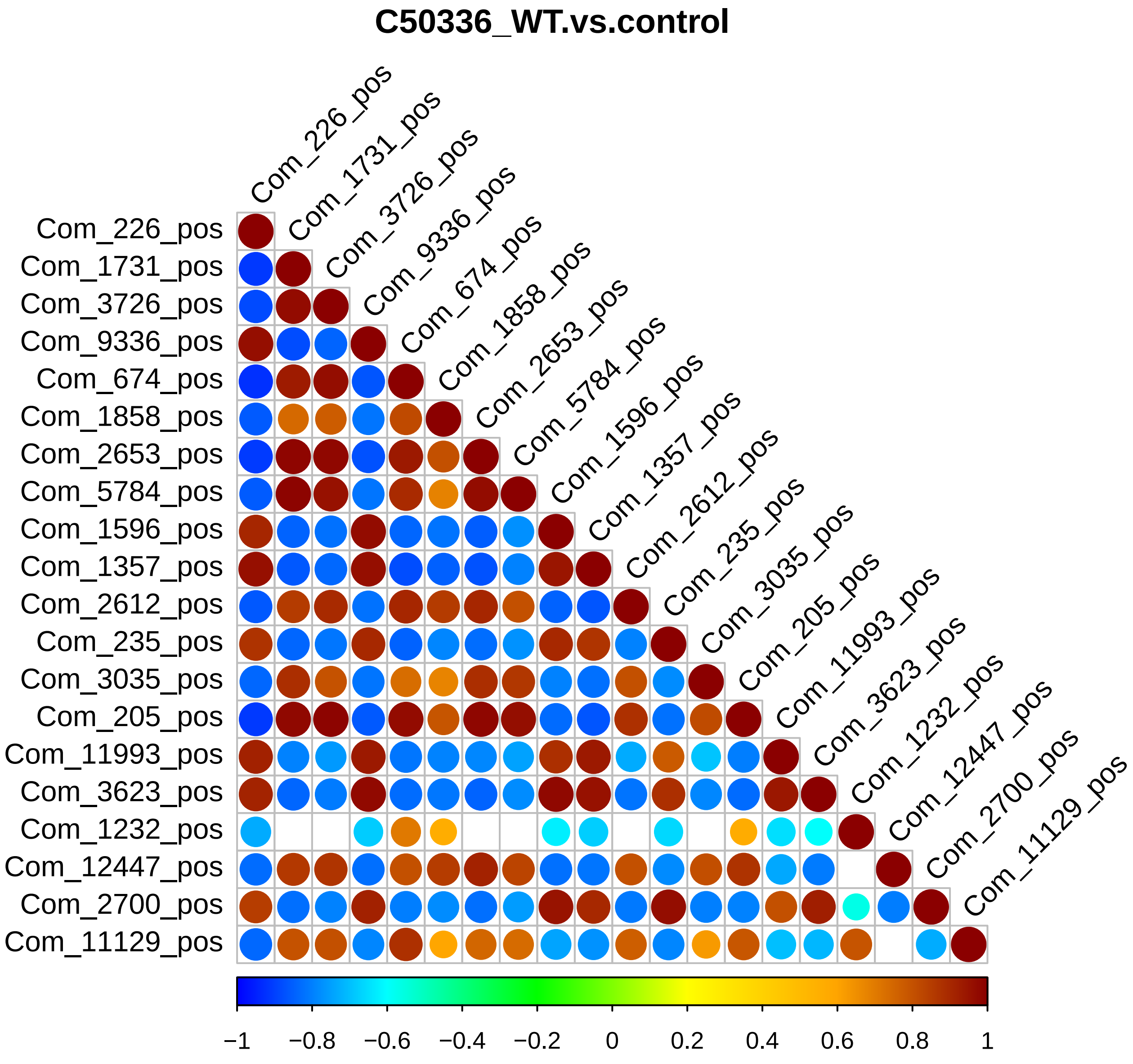
C50336_WT.vs.control
Com_226_pos
Com_1731_pos
Com_3726_pos
Com_9336_pos
Com_674_pos
Com_1858_pos
Com_2653_pos
Com_5784_pos
Com_1596_pos
Com_1357_pos
Com_2612_pos
Com_235_pos
Com_3035_pos
Com_205_pos
Com_11993_pos
Com_3623_pos
Com_1232_pos
Com_12447_pos
Com_2700_pos
Com_11129_pos
−1
−0.8
−0.6
−0.4
−0.2
0
0.2
0.4
0.6
0.8
1
